# Using Theory-Based Frameworks to Identify Barriers and Enablers of Physicians’ Telemedicine Adoption and Develop Intervention Strategies in China: Multicenter Qualitative Study

**DOI:** 10.2196/73412

**Published:** 2025-09-08

**Authors:** Xinxia Wu, Yuting Yang, Yuchan Li, Yuhan Li, Huixian Li, Yanli Lyu, Ke Liu, Tracy Liu, Zheng Hou, Ke Zhang, Xuedong Xu, Changxiao Jin, Yipei Wang

**Affiliations:** 1 Department of Medical Affairs Peking University Third Hospital Beijing China; 2 Office of Internet Hospital Peking University Third Hospital Beijing China; 3 Institute of Hospital Management Peking University Third Hospital Beijing China; 4 Research Center for Standardization of Medical Data Institute for Hospital Management of Tsinghua University Beijing China; 5 School of Public Health Peking University Beijing China; 6 School of Economics and Management Tsinghua University Beijing China; 7 Department of Obstetrics and Gynecology Peking University Third Hospital Beijing China; 8 Department of Otolaryngology Peking University Third Hospital Beijing China; 9 Party Committee Office and Dean’s Office Peking University Third Hospital Beijing China

**Keywords:** telemedicine, Theoretical Domains Framework, Behavior Change Wheel, behavior change techniques, physician, behavior, adoption, qualitative study, China

## Abstract

**Background:**

Telemedicine is developing rapidly, presenting new opportunities and challenges for physicians and patients. Limited research has examined physicians’ behavior during the process of adopting telemedicine and related factors.

**Objective:**

This study aimed to identify perceived barriers and enablers of physicians’ adoption of telemedicine and to develop intervention strategies.

**Methods:**

Three interlinked frameworks, the Theoretical Domains Framework (TDF), the Behavior Change Wheel (BCW), and behavior change techniques (BCTs), were used sequentially to identify the factors for physicians’ telemedicine adoption and to develop intervention strategies. First, guided by the TDF, a questionnaire was developed and administered in interviews with 36 physicians from 29 different medical institutions in Beijing. Second, the content of the semistructured physician interviews was analyzed using the software NVivo 12.0 to extract themes under each domain. Each theme was then classified as either a barrier or an enabler based on the physicians’ language and expression. Third, following the established relationships in the literature, we mapped each domain in the TDF to the corresponding intervention functions and policy categories within BCW. Fourth, for each identified intervention function, we further identified the associated BCTs using the standardized mappings reported in previous studies. The process of identifying intervention functions, policy categories, and techniques was guided by the APEASE (acceptability, practicability, effectiveness, affordability, spill-over effects, and equity) criteria. Last, potential implementation strategies were proposed via focus group discussion.

**Results:**

We identified 50 themes in relation to the adoption of telemedicine. These comprised 27 barriers and 23 enablers, ranging from administrative issues to specific clinical conditions. Of the 14 TDF domains, 11 domains were mentioned. The most frequently mentioned domains were environmental context and resources (10 themes), beliefs about consequences (9 themes), and emotion (7 themes). Major barriers comprised limited acceptance among senior physicians, inconsistent system performance, inadequate platform usability, and inadequate medical insurance coverage. Key enablers included sufficient communication skills and proficiency in system operations, together with the conviction that telemedicine may assist patients with resolving medical issues and prompt support from the IT department when challenges arise. Additionally, 7 of 9 intervention functions, 6 of 7 policy categories, and 26 of 93 BCTs were selected for each theme. Finally, we proposed several potential implementation strategies to enhance physician adoption of telemedicine.

**Conclusions:**

This study identified a range of interventions and strategies that could improve telemedicine adoption in this context. Implementing these measures requires efforts from health administrative departments, medical institutions, and health care personnel.

## Introduction

Telemedicine refers to the use of information and communication technologies to enable the delivery of medical services and clinical interactions between health care providers and patients at different locations [1]. The modes of interaction can be categorized into synchronous and asynchronous formats. Synchronous virtual visits involve real-time interactions between patients and physicians through audio and video communication, while asynchronous services enable non-real-time communication through the transmission of information [2]. Its rapid expansion has contributed to improved distribution of medical resources, enhanced access to health care in remote areas [1,3,4], and reduced health care costs worldwide [5-7]. Many specialties are thriving in high-income countries by providing services through telemedicine. In the United States and United Kingdom, telemedicine services account for approximately 10% to 30% of total encounters [8,9]. In China, by the end of 2024, 3340 internet hospitals had been established nationwide. However, they provided just over 100 million telemedicine services in total [10], accounting for less than 2% of total outpatient services [11]. This low proportion reflects a common challenge faced by many health care institutions—namely, the underutilization of telemedicine services despite the completion of platform development. Contributing factors may include low motivation for physician participation and the imbalance between service supply and patient demand [7,12]. Given the benefits and potentials of telemedicine for promoting the patient experience, enhancing the clinician experience, reducing cost, and improving quality [13,14], it is particularly important for low-income countries to promote its effective use through improved system design and better resource integration, thereby enhancing the sustainability and coverage of telemedicine services [15,16].

The effective implementation of telemedicine fundamentally depends on the active engagement of physicians, who are central to telemedicine delivery. Comprehending how to enhance their willingness to adopt and consistently use this novel service mode is therefore critical. Prior studies have generally used two main approaches to investigate physicians’ acceptance of telemedicine. One approach involves the use of classical models—such as the Technology Acceptance Model, Unified Theory of Acceptance and Use of Technology, and Theory of Planned Behavior—to explore behavioral intentions and their determinants. The other approach relies on regression analyses based on physicians’ demographic characteristics and their volume of telemedicine activities to identify associated factors. Findings from the Technology Acceptance Model suggest that perceived usefulness is a key predictor of physicians’ willingness to adopt telemedicine [17]. The Unified Theory of Acceptance and Use of Technology emphasizes the influence of performance expectancy, effort expectancy, social influence, and facilitating conditions on usage intention [18], while the Theory of Planned Behavior proposes that behavioral intention is jointly determined by attitudes toward the behavior, subjective norms, and perceived behavioral control [19]. Although these models provide relatively comprehensive theoretical frameworks from different perspectives, they do not sufficiently account for a systematic analysis grounded in physicians’ behaviors and cognition. As a result, they offer limited guidance for developing effective strategies and informing practice. Regression-based research has identified factors such as gender, specialty, title, and years of experience with telemedicine services as significantly associated with physicians’ engagement in telemedicine [20], yet many of these factors are difficult to modify in practice. To overcome this constraint, some researchers have turned to theory-based approaches—most notably, the Theoretical Domains Framework (TDF), a framework widely used in behavior change research in health care [21]. The TDF was initially developed in 2005 by Michie and colleagues [22], and it synthesizes 128 theoretical constructs derived from 33 distinct behavior change theories into 14 domains that encapsulate psychological, cognitive, social, and other variables affecting behavior [23,24]. Notably, this framework can systematically identify related factors and provide a theoretical foundation for further intervention [25]. The TDF has been used to inform implementing clinical guidelines [26], infection prevention [27,28], and medication adherence [29].

**Figure 1:**
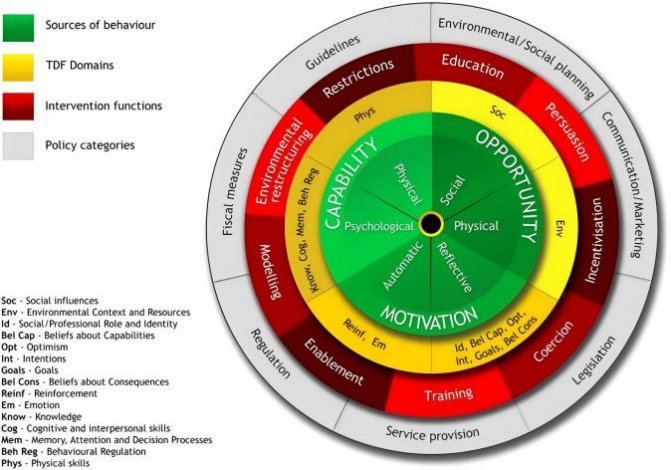
Theoretical Domains Framework (TDF) domains mapped to the Behavior Change Wheel (BCW), showing associations between TDF domains (yellow) and the 9 intervention functions (red) and 7 policy categories (gray) of the BCW. The figure was reproduced with written permission from Michie et al and is protected by copyright.

**Figure 2:**
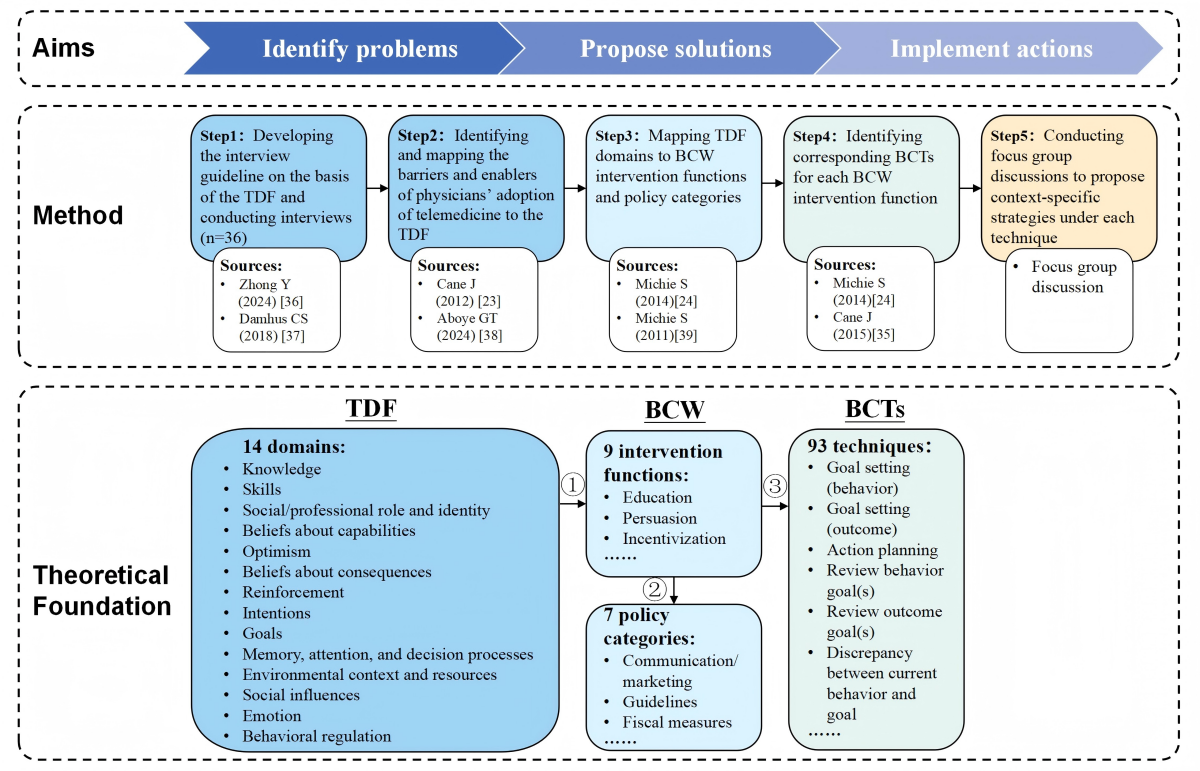
Overview of the study design showing the 5 steps followed in this study, where, in the theoretical foundation, each Theoretical Domains Framework (TDF) domain maps to one or more of the 9 intervention functions of the Behavior Change Wheel (BCW), each intervention function maps to one or more of the 7 policy categories, and each BCW intervention function maps to one or more of the 93 behavior change techniques (BCTs) [23,24,35-39].

Building on the identification of behavioral determinants using the TDF, researchers often integrate TDF with the Behavior Change Wheel (BCW) to convert these findings into intervention functions [30,31]. The BCW is a comprehensive framework that links behavioral determinants to intervention functions and policy categories, providing a structured approach to intervention design [24]. Specifically, the 14 domains of the TDF can be mapped to the 9 intervention functions (eg, education, training) and linked to 7 policy categories (eg, guidelines, service provision) of the BCW, facilitating structured intervention design [22]. See Figure 1 and Figure 2. For example, Forbes et al [28] used the TDF to identify the factors influencing postpartum hemorrhage detection and management, followed by the BCW to guide the selection of appropriate intervention functions like education and training to address this problem. Therefore, integration of TDF and BCW facilitates the translation of abstract behavioral theory into practical, theory-informed intervention development, effectively bridging the gap between identifying problems and designing solutions.

Although the BCW provides a thorough framework for categorizing intervention types and guiding strategic direction, it does not offer specific tools nor techniques for implementation, limiting its practical utility. To mitigate this limitation, researchers frequently combine BCW with behavior change techniques (BCTs), which offer a comprehensive, repeatable array of evidence-based techniques [32]—such as goal setting [33] and feedback [34]—corresponding to each intervention function [35]. The incorporation of BCTs effectively compensates for the BCW’s deficiency in operational specificity, guaranteeing that intervention strategies are not only theoretically grounded and structurally organized but also practically implementable.

This multicenter qualitative study used an established rationale for the TDF, BCW, and BCT frameworks to examine the barriers and enablers affecting physicians’ adoption of telemedicine in China. By linking behavioral determinants to intervention functions and specifying actionable techniques, this study offers a robust foundation for developing targeted strategies to promote telemedicine uptake and address challenges with expanding health care access in developing contexts. The TDF, BCW, and BCT frameworks provide a structured approach for identifying problems, proposing solutions, and implementing actions. This framework analysis enhances the understanding of both individual-level and contextual factors, offering a theory-based practical case. These findings are expected to contribute to the global evolution of telemedicine and patient-centered services, providing actionable insights for future research and practice.

## Methods

### Ethical Considerations

Ethics approval was granted by the Medical Science Research Ethics Committee of Peking University Third Hospital (IRB00006761-M2024300). The participants provided informed consent. To ensure confidentiality, all interview data were anonymized and securely stored on password-protected, access-restricted institutional servers. Only the research team had access to the data. Participants received compensation of ¥200 (US $27.85) as a token of appreciation for their participation.

### Design and Setting

This study aimed to identify problems, propose solutions, and implement actions, which was conducted in 5 steps (Figure 2): (1) developing the interview guideline on the basis of the TDF and conducting interviews [36,37]; (2) identifying the themes mentioned in the interview, classifying them into barriers and enablers, and mapping these barriers and enablers of physicians’ adoption of telemedicine to the relevant TDF domains [23,38]; (3) mapping each TDF domain to its corresponding BCW intervention functions and policy categories, following established literature [24,39], which enables the translation of findings from behavioral analysis into actionable strategies; (4) identifying corresponding BCTs for each intervention function based on published mappings to obtain operationalizable and replicable techniques [24,35]; and (5) conducting focus group discussions to propose context-specific strategies for each technique to support increased physician uptake of telemedicine. This study is reported in accordance with the Consolidated Criteria for Reporting Qualitative Research [40] (COREQ; Multimedia Appendix 1).

### Step 1: Developing the Interview Guideline on the Basis of the TDF and Conducting Interviews

#### Study Setting and Institutional Sampling

We performed a survey in a metropolitan city (Beijing) that plays a leading role in developing telemedicine in China. We included tertiary and nontertiary hospitals (comprising level I and level II hospitals and clinics) that provide telemedicine. Both public and private medical institutions were covered. To ensure diversity in institutional characteristics and practice experience, we selected medical institutions with varying levels of telemedicine service volume. The sample comprised institutions with an average annual telemedicine volume of more than 10,000 consultations, ranging from 1000 to 10,000 consultations, and fewer than 1000 consultations. That ensured the inclusion of institutions with different service capacities and development stages in telemedicine, enhancing the comprehensiveness and applicability of the findings.

#### Participant Recruitment and Inclusion Criteria

Based on that, we used purposive sampling to select physicians who had conducted both live or synchronous video and asynchronous graphic consultations on institution-built telemedicine platforms. Each medical institution included one to several participating physicians. Simultaneously, we considered physicians’ sex, age, department, and other relevant characteristics to ensure that the sample was representative of the physician population within the participating medical institutions. This diversity guaranteed a comprehensive representation of professional viewpoints. The inclusion criteria were as follows: (1) ≥3 years of work experience; (2) provided telemedicine services for ≥6 months; (3) was willing to voluntarily participate in this interview; and (4) possessed good communication skills, with the ability to clearly express their thoughts and experiences during in-depth qualitative interviews.

#### Development and Pilot Testing of the Interview Guide

We used the TDF framework in the design, data collection, and analysis of the qualitative investigation of the implementation. The TDF informed the interview guide, with 1 to 2 questions formulated to explore each of the 14 domains. For example, one of the questions was “What do you know about telemedicine?” Investigators with expertise in implementation science provided guidance to develop the interview guide, which was further refined through consultations with experts in hospital management and health policy management as well as physicians. We conducted 2 pre-experiments after the outline was initially finalized. The interview outline was refined slightly after the pretests, and the samples from the pretests were not included in the study owing to slight changes. Table 1 presents the definitions of the theoretical domains and the key interview questions aligned with the TDF domains. The original Chinese version of the interview guide is provided in Multimedia Appendix 2 for interested readers.

**Table 1:** Definitions of the theoretical domains and the key interview questions aligned with the Theoretical Domains Framework (TDF).

TDF domains	Definition	Interview questions
Knowledge	An awareness of the existence of something	What do you know about telemedicine? What do you think are appropriate/inappropriate for telemedicine?
Skills	An ability or proficiency acquired through practice	Do you know how to perform telemedicine? Which skills do you think physicians need when using telemedicine? Are you good at it?
Social/professional role and identity	A coherent set of behaviors and displayed personal qualities of an individual in a social or work setting	Will different types of physicians have different needs for telemedicine? For example, physicians of different ages, genders, and degrees
Beliefs about capabilities	Acceptance of the truth, reality, or validity about an ability, talent, or facility that a person can put to constructive use	To what extent do you feel capable of conducting telemedicine? When you conduct telemedicine, is there someone to assist you? If so, who and how?
Optimism	The confidence that things will happen for the best or that desired goals will be attained	Do you believe telemedicine can make a difference for patients? Will you increase, decrease, or maintain the percentage of telemedicine in your overall health care in the future, and why?
Beliefs about consequences	Acceptance of the truth, reality, or validity about outcomes of a behavior in a given situation	What are the advantages of telemedicine? What are the disadvantages of telemedicine?
Reinforcement	Increasing the probability of a response by arranging a dependent relationship, or contingency, between the response and a given stimulus	Which factors can motivate you to perform telemedicine?
Intentions	A conscious decision to perform a behavior or resolve to act in a certain way	Are you willing to carry on with telemedicine？ Do you think telemedicine can solve patients’ problems？
Goals	Mental representations of outcomes or end states that an individual wants to achieve	What can you achieve when performing telemedicine?
Memory, attention, and decision processes	The ability to retain information, focus selectively on aspects of the environment, and choose between two or more alternatives	Are there elements that are difficult to remember, pay attention to, and make decisions about in telemedicine?
Environmental context and resources	Any circumstance of a person’s situation or environment that discourages or encourages the development of skills and abilities, independence, social competence, and adaptive behavior	What environment and resources will the medical institution or department provide for telemedicine? To what extent has it supported the successful implementation?
Social influences	Those interpersonal processes that can cause individuals to change their thoughts, feelings, or behaviors	Which attitudes do your colleagues and institution hold about telemedicine? Does their opinion influence the way you adopt telemedicine? Is there any difference between the supportive and non-supportive groups? And what are the differences?
Emotion	A complex reaction pattern, involving experiential, behavioral, and physiological elements, by which the individual attempts to deal with a personally significant matter or event	What are your emotions like when you use telemedicine?
Behavioral regulation	Anything aimed at managing or changing objectively observed or measured actions	Which changes are necessary for telemedicine to succeed? How to make the telemedicine process easier and smoother？

#### Data Collection and Management Procedures

In-depth face-to-face interviews were conducted from April 2024 to July 2024 with physicians who used telemedicine. All participants provided verbal informed consent. The interviews were conducted in Mandarin Chinese by the authors XW, YY, and Yuchan Li. All interviewers received professional training in qualitative interviewing and had extensive experience conducting qualitative research (n=3, women, health management practitioners). Before the interview, the interviewer gave a brief self-introduction and overview of the interview content to establish the relationship with the interviewee. Each interview was conducted by 1 or 2 of the 3 trained interviewers (XW, YY, and Yuchan Li), depending on their scheduling. We recorded the interviews using a Sogou digital voice recorder. Data saturation was reached when the data collection yielded no new information about the barriers and facilitators influencing the adoption of telemedicine services. Finally, 36 participants were included in this study. The interviews ranged in length from 18 minutes to 41 minutes (mean 28 minutes; median 25 minutes). The recordings were transcribed into textual materials within 48 hours after the interviews, and the researcher personally interviewed, recorded, and sorted the data to ensure consistency. The participants were allocated a randomly selected unique identification number.

### Step 2: Identifying and Mapping the Barriers and Enablers of Physicians’ Adoption of Telemedicine to the TDF

#### Transcription and Data Coding

We transcribed and organized the interview transcripts prepared for the data coding. We adopted the software NVivo 12.0 to code the physician’s interview data and distill the main viewpoint. Qualitative data were analyzed using the 7-step analysis by Colaizzi [41]: (1) read all interview transcripts carefully; (2) extract statements of significance; (3) code recurring ideas; (4) summarize coded ideas; (5) write a detailed, omission-free description; (6) identify similar ideas and summarize themes; and (7) return to interviewees for evidence. We reached out to 5 participants through email or phone and provided a summary of the detected themes and interpretations following the completion of the research. Participants were solicited to evaluate the findings and offer input about the extent to which the results accurately represented their experiences and viewpoints.

#### Mapping Themes to the TDF Domains

Data analysis began with inductive coding to identify the barriers and enablers that arose from the data, followed by mapping the factors to the TDF. We analyzed the textual material via content analysis and inductive methods [42], and the text was coded into the theoretical domain that best reflects the key topic [30,43]. To be specific, the 14 domains of the TDF previously described were used as the coding framework [44]. Second, we used an inductive coding approach to group similar points to form themes within the initial coding scheme of the 14 TDF domains. For example, *feeling relaxed with less workload* was an enabler coded to *emotion*. Finally, we further examined these themes, and each theme was then classified as either a barrier or an enabler based on the physicians’ expression. To prepare the results, we tabulated the themes into barriers and enablers separately.

#### Research Team Consensus and Coding Reliability

Two of the authors (XW and YY) analyzed the data independently. After completing the coding, both authors compared the coding selections, and when discrepancies occurred, consensus was reached through discussion with a third author (YW) until agreement was reached.

### Step 3: Mapping TDF Domains to BCW Intervention Functions and Policy Categories

The BCW is a comprehensive framework for designing behavior change interventions by systematically linking behavioral determinants to intervention functions and policy categories [24,28]. According to Michie et al [24], the literature delineates these connections, whereby each domain of the TDF can be mapped to one or more BCW intervention functions, which are subsequently mapped to relevant policy categories.

### Step 4: Identifying Corresponding BCTs for Each BCW Intervention Function

The BCTs provide more standardized and detailed intervention components, encompassing 93 evidence-based techniques that can be mapped to the 9 intervention functions of the BCW to inform the selection of targeted strategies [32] (Table 3.3 in Multimedia Appendix 3). The literature suggests that a wide array of BCTs may be considered for each intervention function, generally prioritizing the more frequently used techniques.

**Figure 3:**
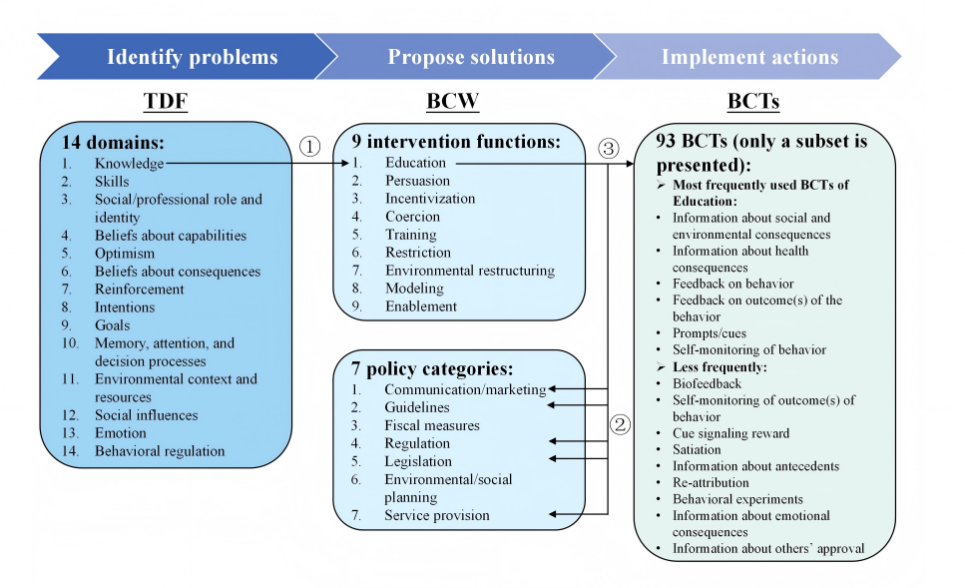
Sequential mapping from the Theoretical Domains Framework (TDF) to the Behavior Change Wheel (BCW) and behavior change techniques (BCTs) to guide intervention design using the knowledge domain as an example.

An example is shown in Figure 3 for greater clarification of the process. In Step 1, if a barrier or enabler was coded in the *knowledge* domain*,* it was mapped to the *education* intervention function (Table 2.2 in Multimedia Appendix 3)*.* In Step 2, the *education* intervention function was subsequently mapped to relevant policy categories, including *communication/marketing, guidelines, regulation, legislation,* and *service provision*, with one or more suitable options selected based on the contextual needs (Table 2.9 in Multimedia Appendix 3). In Step 3, BCTs were selected based on the established association with the *education* intervention function that was prioritized from those most commonly associated with educational strategies, including *information about social and environmental consequences, information about health consequences, feedback on behavior, feedback on outcome(s) of the behavior, prompts/cues,* and *self-monitoring of behavior* (Table 3.3 in Multimedia Appendix 3)*.* If none of the commonly used BCTs were deemed appropriate given the contextual constraints, less frequently used but still functionally relevant BCTs were considered as alternatives. The complete English version of the BCT Taxonomy v1 is provided in Multimedia Appendix 4 [32]. In Steps 2 and 3, we adhered to the APEASE (acceptability, practicability, effectiveness, affordability, spill-over effects, and equity) criteria [24,45], drew upon prior literature, and used focus group discussion to identify the intervention function and policy categories. Our group members included clinical physicians who provide telemedicine services, staff of the Office of Internet Hospital, and experts in related fields.

### Step 5: Conducting Focus Group Discussions to Propose Context-Specific Strategies for Each Technique

Based on the results of Steps 1 to 4, examples of potential ways to implement these intervention strategies were generated from prior literature and combined focus group discussions, enhancing their potential for successful implementation in clinical practical.

## Results

### Characteristics of Institutions and Physicians

The 36 physicians came from 29 different medical institutions in Beijing, with 1 to 8 physicians from each institution. The majority were public institutions (22/29, 76%), and most were tertiary-level facilities (22/29, 76%). Regarding service volume, more than one-half of the institutions (16/29, 55%) had an annual offline service volume exceeding 1,000,000 visits. In terms of volume of telemedicine services, 45% (13/29) of the institutions provided 10,000 to 100,000 telemedicine services annually. As for the participants, most physicians were from public (29/36, 81%) and tertiary institutions (30/36, 84%). There were more women (21/36, 58%) than men (15/36, 42%). The physicians ranged in age from 31 years to older than 60 years; most (18/36, 50%) had doctoral degrees, and the largest percentage had deputy chief physician titles (21/36, 58%). Physicians were from internal medicine (ie, cardiology, endocrinology, and metabolism), surgery (ie, general surgery, orthopedics), obstetrics and gynecology, anesthesiology, and others. They mainly had 1 year to 3 years of telemedicine work (21/36, 58%). Table 2 shows the details.

**Table 2:** Characteristics of institutions and physicians.

Characteristics				Values, n (%)
**Characteristics of institutions (n=29)**				
	**Institution type**			
		Public	22 (76)	
		Private	7 (24)	
	**Institution level**			
		Tertiary	22 (76)	
		Nontertiary	7 (24)	
	**Annual volume of offline services (visits)**			
		<100,000	5 (17)	
		100,000-1,000,000	8 (28)	
		>1,000,000	16 (55)	
	**Annual volume of telemedicine services (visits)**			
		<10,000	12 (41)	
		10,000-100,000	13 (45)	
		>100,000	4 (14)	
**Characteristics of physician (n=36)**				
	**Gender**			
		Male	15 (42)	
		Female	21 (58)	
	**Age (years)**			
		31-40	11 (31)	
		41-50	19 (52)	
		51-60	3 (8)	
		>60	3 (8)	
	**Education**			
		Doctor	18 (50)	
		Master	11 (31)	
		Bachelor	7 (19)	
	**Title**			
		Chief physician	7 (19)	
		Deputy chief physician	21 (58)	
		Attending physician	8 (22)	
	**Department**			
		Internal Medicine	20 (56)	
		Surgery	6 (17)	
		Obstetrics and Gynecology	3 (8)	
		Anesthesiology	2 (6)	
		Psychiatry and Mental Health	2 (6)	
		Pediatrics	1 (3)	
		Otolaryngology	1 (3)	
		Sports Medicine	1 (3)	
	**Experience in telemedicine service (years)**			
		<1	4 (11)	
		1-3	21 (58)	
		≥4	11 (31)	

### Physicians’ Barriers to and Enablers of Telemedicine Aligned With the TDF

**Figure 4:**
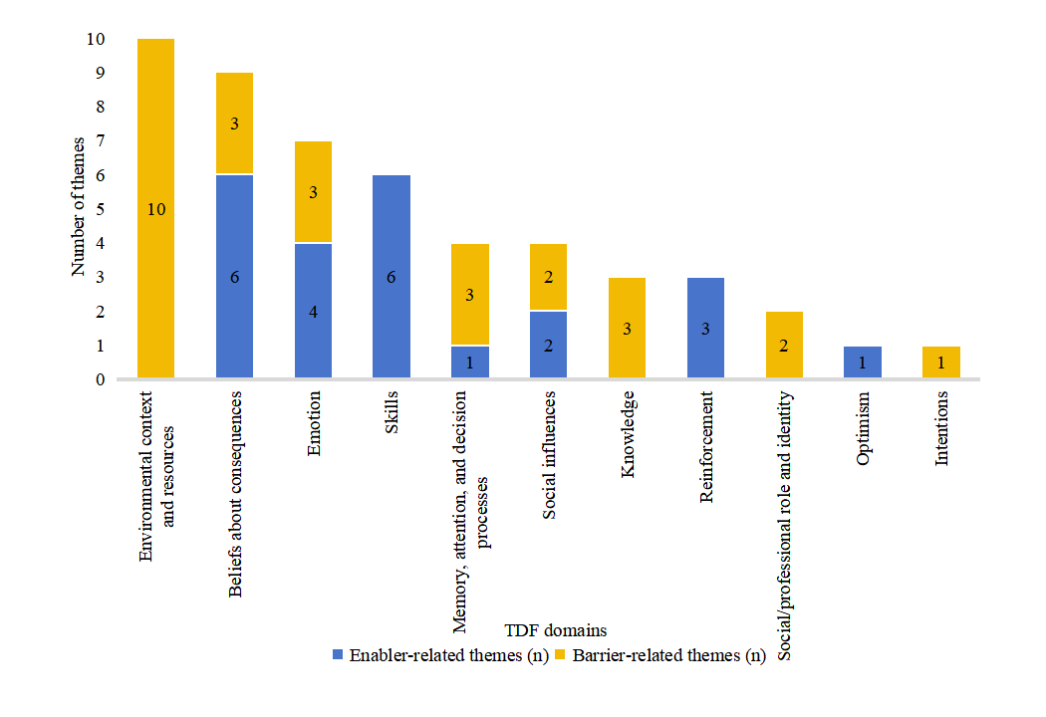
The frequencies of barrier- and enabler-related themes across the Theoretical Domains Framework (TDF) domains.

A total of 50 themes were identified in relation to the adoption of telemedicine, comprising 27 barriers and 23 enablers and encompassing 11 of the 14 TDF domains. No viewpoint aligned with the goals, beliefs about capabilities, and behavioral regulation domains; we received relevant responses to the pertinent interview questions. Throughout the coding and analysis, the replies were more appropriately categorized under other TDF domains. The barriers referred to 8 of the 14 domains, and enablers were involved in 7 of the 14 domains (4 domains had both barriers and enablers). See Figure 4. Tables 3 and 4 also present the themes and frequencies of the domains.

**Table 3:** Barrier-related themes to physicians’ (n=36) adoption of telemedicine.

Domains and themes			Responses, n
**Environmental context and resources**			
	Inconsistent system performance	14	
	Inadequate platform usability	13	
	Inadequate medical insurance payment mechanisms	13	
	Network delay	11	
	Imperfect management mechanisms that lacked overall coordination and support	8	
	Imbalance in workload between online and offline practice affecting the implementation of telemedicine	8	
	No well-established pricing mechanism for telemedicine services	7	
	Electronic medical records from other medical institutions inaccessible by different health care providers	5	
	Lack of dedicated telemedicine venues	5	
	Restricted scope of telemedicine services	2	
**Knowledge**			
	Limited awareness and familiarity with the app’s functionalities among some physicians	12	
	The applicability of telemedicine dependent on the characteristics of the clinical specialties	12	
	Patients encountering operational challenges with mobile devices or systems, which adversely affect physician-patient interactions	8	
**Memory, attention, and decision processes**			
	Inefficiencies caused by turnaround times in asynchronous consultations	12	
	Patients exhibiting limited ability to articulate their medical conditions, which, to some extent, impacts the accuracy of the physician's assessment	2	
	Consultations sometimes conducted by family members on behalf of patients, leading to potential information biases	1	
**Beliefs about consequences**			
	Online communication adversely affecting the physician-patient experience during telemedicine services	12	
	Telemedicine services associated with potential risks to medical quality and safety	10	
	Low efficiency in online interactions and consultations between physicians and patients	8	
**Emotion**			
	Concerns regarding privacy breaches and the risk of disputes	6	
	Anxiety stemming from fragmented communication that precludes real-time responses	4	
	Sense of detachment fostered by an inability to see the patient	1	
**Social influences**			
	Lack of collaboration between health care professionals	9	
	Insufficient availability of professional customer service personnel to address nonmedical logistic issues	5	
**Social or professional role and identity**			
	Limited acceptance among senior physicians	16	
	Limited willingness among physicians with senior titles	5	
**Intentions**			
	Telemedicine consultations scheduled at the department level rather than based on individual physician preferences	1	

**Table 4:** Enabler-related themes for physicians’ (n=36) adoption of telemedicine.

Domains and themes		Responses, n
**Skills**		
	Communication skills	20
	System operation skills	13
	Clinical capabilities	9
	Information-gathering skills	5
	Adaptability	4
	Clinical experience	3
**Beliefs about consequences**		
	Improve the convenience and accessibility of medical service	11
	Guarantee the continuity of medical service	5
	Reduce the patient burden	5
	Improve physicians’ personal influence	4
	Attract more patients through the online platform	4
	Facilitate physicians’ management of patients	3
**Emotion**		
	A sense of accomplishment derived from being able to assist patients with resolving their issues	12
	Feel relaxed with less workload	8
	Telemedicine work environment being conducive to maintaining a calm and composed mindset for physicians	4
	Enjoying the convenience of online medical work	4
**Reinforcement**		
	Increases the proportion of online workload assessments	3
	Links online work evaluation to the promotion of professional titles	2
	Links patient evaluation to work assessment	2
**Social influences**		
	The IT department prompts support when encountering issues with the operation of the information system	17
	Both medical institutions and colleagues actively support the implementation of telemedicine services	5
**Memory, attention, and decision processes**		
	Telemedicine enabling real-time collection of patient information, which can be analyzed by artificial intelligence (AI) to support clinical decision-making	1
**Optimism**		
	Conviction that telemedicine may assist patients with resolving medical issues and willing to devote more effort	15

### Barriers

#### Environmental Context and Resources

Available resources and the environment influence a physician’s inclination to perform [46]. Environmental context and resources have emerged as key barriers to using telemedicine. There were 10 barrier-related themes in this domain (in order of frequency): inconsistent system performance, inadequate platform usability, inadequate medical insurance payment mechanisms, network delay, imperfect management mechanisms that lacked overall coordination and support, imbalance in workload between online and offline practice affects the implementation of telemedicine, no well-established pricing mechanism for telemedicine services, electronic medical records from other medical institutions that cannot be accessed by different health care providers, lack of dedicated telemedicine venues, and restricted scope of telemedicine services (Table 3). These 10 themes accounted for 37% (10/27) of the total barriers. They can be further categorized into technical issues and inadequate management mechanisms.

The functions are relatively simple and support only one type of audio, video, or text. It is better to cover audio and video as well as add other additional functions.

Dr A

The restrictions on telemedicine are too conservative and could be significantly loosened. The current restrictions on follow-up consultations are very strict.

Dr B

Telemedicine service fees are lower, the remote assessment, rehabilitation guidance, and nursing consultation are free, resulting in insufficient revenue and compliance.

Dr C

#### Knowledge

Knowledge is important because a physician’s perceived awareness of the rationale, procedure(s), and task environment associated with a desired behavior is likely to affect whether a physician implements it [46]. There were 3 barrier-related themes in this domain (in order of frequency): limited awareness and familiarity with the app’s functionalities among some physicians; the applicability of telemedicine being dependent on the characteristics of the clinical specialties; and patients encountering operational challenges with mobile devices or systems, which adversely affect physician-patient interactions (Table 3). These 3 themes accounted for 11% (3/27) of the total barriers.

Medical institutions should actively guide and increase publicity to enable physicians and patients to fully understand the benefits and related functions of telemedicine services.

Dr D

#### Memory, Attention, and Decision Processes

Remembering to enact a particular behavior or remaining focused on it and the ability to choose between one or more alternatives are likely to affect whether the behavior is implemented [46]. There were 3 barrier-related themes in this domain (in order of frequency): inefficiencies caused by turnaround times in asynchronous consultations; patients exhibiting limited ability to articulate their medical conditions, which, to some extent, impacts the accuracy of the physician’s assessment; and consultations sometimes conducted by family members on behalf of patients, leading to potential information bias (Table 3). These 3 themes accounted for 11% (3/27) of the total barriers.

Telemedicine involves no real-time communication, and the interaction is delayed. I might forget to reply to patients.

Dr E

#### Beliefs About Consequences

The beliefs a physician holds about the outcomes of a particular behavior affect whether they decide to comply [46]. There were 3 barrier-related themes in this domain (in order of frequency): online communication adversely affecting the physician-patient experience during telemedicine services, telemedicine services associated with potential risks to medical quality and safety, and low efficiency in online interactions and consultations between physicians and patients (Table 3). These 3 themes accounted for 11% (3/27) of the total barriers.

The external characteristics of the patient cannot be seen online, which prevents timely detection of changes in the patient’s condition and may pose a certain safety risk.

Dr C

We often spend more time when conducting telemedicine, as often need to review previous medical records, write prescriptions, interpret reports, and we also follow-up interactions.

Dr F

#### Emotion

Emotion is associated with a desired behavior and is likely to affect whether a physician decides to perform it [46]. There were 3 barrier-related themes in this domain (in order of frequency): concerns regarding privacy breaches and the risk of disputes; anxiety stemming from fragmented communication that precludes real-time responses; and a sense of detachment fostered by the inability to see the patient (Table 3). These 3 themes accounted for 11% (3/27) of the total barriers.

We always need to swipe the mobile phone to see if there is a reply from the patient. Some patients will ask us to reply as soon as possible, and this kind of will lead to anxiety.

Dr G

#### Social Influences

Interpersonal processes that can cause physicians to change their thoughts, feelings, or behaviors can often influence the performance of a desired behavior [46]. There were 2 barrier-related themes in this domain (in order of frequency): lack of collaboration between health care professionals and insufficient availability of professional customer service personnel to address nonmedical logistic issues (Table 3). These 2 themes accounted for 7% (2/27) of the total barriers.

There are no medical assistants or secretaries when conducting telemedicine, and physicians’ energy cannot fully devoted to most core aspects.

Dr A

#### Social or Professional Role and Identity

The degree to which physicians believe that a particular behavior aligns with their social or professional identity is likely to influence whether they will implement it [46], and the difference is reflected mainly in the different characteristics of individuals (eg, age, education) [47]. There were 2 barrier-related themes in this domain (in order of frequency): limited acceptance among senior physicians and limited willingness among physicians with senior titles (Table 3). These 2 themes accounted for 7% (2/27) of the total barriers.

Senior physicians are less likely to be as enthusiastic about telemedicine as younger physicians are. They are also pressed with ward inspections, directed students, conducted research, and other things.

Dr D

#### Intentions

The level of motivation a physician has or the commitment that they make to act in a particular way is likely to affect whether they do so [46]. There was 1 barrier-related theme in this domain: telemedicine consultations scheduled at the department level rather than based on individual physician preferences (Table 3). This theme accounted for 3.7% (1/27) of the total barriers.

Telemedicine work is scheduled by the department, not my individual preferences.

Dr H

I hope the medical institution does not put too much pressure on physicians, allowing us to work within our abilities, and not make it mandatory.

Dr G

### Enablers

#### Skills

Skills are thought to be important determinants of behavior change because a physician’s perceived sense of their own competence in performing a desired behavior is likely to affect whether they are willing and able to implement it [46]. There were 6 enabler-related themes in this domain (in order of frequency): communication skills, system operation skills, clinical capabilities, information-gathering skills, adaptability, and clinical experience (Table 4). These 6 themes accounted for 26% (6/23) of the total enablers. Several physicians expressed that sufficient communication skills and proficiency in system operations were necessary when conducting telemedicine and that they possessed it.

Physicians cannot perform patients’ physical examinations when conducting telemedicine services, resulting in limited information obtained. Therefore, it is necessary for physicians to have excellent communication skills and humanistic qualities. And I think I have good communication skills.

Dr I

#### Beliefs About Consequences

Concerning beliefs about consequences, there were 6 enabler-related themes in this domain (in order of frequency): improve the convenience and accessibility of medical service, guarantee the continuity of medical service, reduce the patient burden, improve physicians’ personal influence, attract more patients through the online platform, and facilitate physicians’ management of patients (Table 4). These 6 themes accounted for 26% (6/23) of the total enablers. The results can be classified into two aspects: an altruistic perspective and a selfish perspective. From an altruistic perspective, physicians believed that telemedicine could improve the convenience and accessibility of medical services, guarantee the continuity of medical services, and reduce patient burden. From a selfish perspective, physicians considered that telemedicine can improve physicians’ personal influence, attract more patients through the online platform, and facilitate physicians’ management of patients.

Majority of our patients were from other cities, possibly over 60%. We often take medical histories, perform physical examinations, and conduct laboratory tests during offline visits. Then, they advised to consult test results online.

Dr J

Telemedicine is a way to build a physician brand and increase patient stickiness.

Dr A

#### Emotion

Positive emotions are likely to increase physicians’ motivation to conduct telemedicine [46]. There were 4 enabler-related themes in this domain (in order of frequency): a sense of accomplishment derived from being able to assist patients with resolving their issues, feeling relaxed with less workload, the telemedicine work environment being conducive to maintaining a calm and composed mindset for physicians, and enjoying the convenience of online medical work (Table 4). These 4 themes accounted for 17% (4/23) of the total enablers.

I think telemedicine can help patients solve most problems, and I feel accomplished for that.

Dr K

Telemedicine is more relaxed and peaceful than offline consultations are, and I feel less stressed.

Dr L

#### Reinforcement

Reinforcement is believed to be important because the perceived rewards and punishments associated with the performance or nonperformance of a particular behavior are likely to affect whether someone decides to implement it [46]. There were 3 enabler-related themes in this domain (in order of frequency): increases the proportion of online workload assessments, links online work evaluation to the promotion of professional titles, and links patient evaluations to work assessments (Table 4). These 3 themes accounted for 13% (3/23) of the total enablers.

It is not enough just to say supporting telemedicine; taking some specific regulations is necessary. For example, link online work to the promotion of professional titles. I believe that will increase the initiative of departments and physicians.

Dr D

#### Social Influences

Factors such as support from others can often influence the performance of a desired behavior [46]. There were 2 enabler-related themes in this domain (in order of frequency): The IT department prompts support when encountering issues with the operation of the information system, and both medical institutions and colleagues actively support the implementation of telemedicine services (Table 4). These 2 themes accounted for 9% (2/23) of the total enablers.

If we encounter any problems, we usually report them to the staff of the information center, who can resolve these problems quickly.

Dr I

#### Memory, Attention, and Decision Processes

There was 1 enabler-related theme in this domain: Telemedicine enables the real-time collection of patient information, which can be analyzed using artificial intelligence to support clinical decision-making (Table 4). This theme accounted for 4% (1/23) of the total enablers.

With the development of AI, telemedicine can greatly assist physicians in collecting patient information. Physicians cannot guarantee that they remember all the knowledge accurately and comprehensively, and they cannot access this information in real time offline. However, telemedicine provided some prompts to help physicians make decisions and review their work.

Dr M

#### Optimism

The extent to which a physician believes a goal will be achieved will affect the likelihood of them performing the behavior(s) that will lead to that goal [46]. There was 1 enabler-related theme in this domain: conviction that telemedicine may assist patients with resolving medical issues and willing to devote more effort (Table 4). This theme accounted for 4% (1/23) of the total enablers.

Telemedicine can solve most patients' problems and reach 80–90% of the offline level, which is meaningful.

Dr N

### Identified Corresponding Intervention Functions, Policy Categories, BCTs, and Implementation Strategies for Each Barrier and Enabler

We provided relative intervention functions, policy categories, and strategies for each barrier and enabler. There were 7 of 9 intervention functions, 6 of 7 policy categories, and 26 of 93 BCTs selected. No barrier nor enabler mapped to the intervention functions of restriction and modeling nor mapped to the policy categories of legislation. We retained two TDF domains that involved the most barrier and enabler themes, along with the corresponding intervention functions, policy categories, and proposed strategies in Table 5. For instance, the barrier of no well-established pricing mechanism (inadequate medical insurance payment mechanisms) was identified in the *environmental context and resources* domain. To address this, the *enablement* intervention function was selected, as it is suited to overcoming external constraints and facilitating behavior change by removing barriers or increasing means or resources. To effectively deliver this function, the *fiscal measures* policy categories were identified as the most suitable channels. Subsequently, the relevant BCTs of “1.3 Goal setting (outcome)” and “3.2 Social support (practical)” were selected to operationalize this strategy. Accordingly, a proposed implementation strategy was setting pricing standards and adjusting the charging scope, linking professional titles to medical service fees. Multimedia Appendix 5 provides the complete results.

**Table 5:** Identified corresponding intervention functions, policy categories, behavior change techniques (BCTs), and implementation strategies for each barrier and enabler (brief version).

TDF^a^ domains and themes of the barriers and enablers to use telemedicine (abbreviated version)			Intervention functions		Policy categories		BCTs		Proposed implementation strategies
**Environmental context and resources**									
	No well-established pricing mechanism (B^b^)	Enablement		Fiscal measures		1.3 Goal setting (outcome), 3.2 Social support (practical)		Set pricing standards and adjust the charging scope: link professional titles to medical service fees	
	Inadequate medical insurance mechanisms (B)	Enablement		Fiscal measures		1.3 Goal setting (outcome), 3.2 Social support (practical)		Increase financial investment to promote real-time and cross-region settlement functionalities for medical insurance	
	Restricted scope of telemedicine services (B)	Enablement		Regulation		1.1 Goal setting (behavior), 1.3 Goal setting (outcome)		Incorporate more suitable online consultation services, such as nursing consultation fees	
	Imperfect medical institution management mechanism (B)	Enablement		Regulation		1.1 Goal setting (behavior), 1.3 Goal setting (outcome)		Strategically allocate resources such as personnel, finances, and materials to coordinate telemedicine services	
	Imbalanced workload between online and offline (B)	Enablement		Regulation		1.1 Goal setting (behavior), 1.3 Goal setting (outcome)		Establish reasonable online and offline work objectives for physicians to enact a balanced distribution of tasks	
	EMRs^c^ not accessible across different institutions (B)	Environment restructuring		Environmental or social planning		12.1 Restructuring the physical environment, 12.5 Adding objects to the behavior		Boost technical or financial support to strive for cross-regional sharing of EMRs	
	Inconsistent system performance (B)	Environment restructuring		Environmental or social planning		12.1 Restructuring the physical environment, 12.5 Adding objects to the behavior		Boost technical support to enhance system functionality and environmental reconstruction or optimization	
	Network delay (B)	Environment restructuring		Environmental or social planning		12.1 Restructuring the physical environment, 12.5 Adding objects to the behavior		Boost technical support to optimize network environment	
	Inadequate platform usability (B)	Environment restructuring		Environmental or social planning		12.1 Restructuring the physical environment, 12.5 Adding objects to the behavior		Boost technical support to enhance platform functionality diversity, focus on age-appropriate adaptations	
	Lack of telemedicine venues (B)	Environment restructuring		Environmental or social planning		12.1 Restructuring the physical environment, 12.5 Adding objects to the behavior		Boost financial support to rebuild the physical environment to provide specialized telemedicine facilities	
**Beliefs about consequences**									
	Potential risks to medical quality (B)	Education		Guidelines		4.4 Behavioral experiments, 4.1 Instructions on how to perform the behavior		Collect online adverse events and provide recommendations on how to ensure medical safety and quality	
	Low efficiency in online interactions (B)	Education		Guidelines		6.1 Demonstration of the behavior		Provide customized response templates to physicians that improve the efficiency of the physician-patient interaction	
	Affecting the physician-patient experience (B)	Education		Communication or marketing		13.2 Framing/reframing		Propose practices that can improve the physician-patient experience	
	Improve the accessibility of medical services (E^d^)	Education		Communication or marketing		9.1 Credible source, 6.1 Demonstration of the behavior		Use authoritative information sources to provide references or imitations for physicians	
	Guarantee the continuity of medical services (E)	Education		Communication or marketing		6.1 Demonstration of the behavior, 8.3 Habit formation		Demonstrate to physicians the benefits of telemedicine for continuous care and recommend using it to develop “habitual behaviors”	
	Improve physicians’ influence (E)	Education		Communication or marketing		6.2 Social comparison, 6.3 Information about others’ approval		Display to physicians the performance metrics of successful telemedicine using individuals and compare these metrics with the physician’s personal performance	
	Attract more patients (E)	Education		Communication or marketing		9.1 Credible source, 6.1 Demonstration of the behavior		Use authoritative information sources to inform physicians that compelling patient attraction is achievable through online channels	
	Facilitate physicians’ management of patients (E)	Education		Communication or marketing		6.2 Social comparison, 6.3 Information about others’ approval		Demonstrate to physicians the practices and advantages of online patient management, comparing these with the physician’s own	
	Reduce the patient burden (E)	Education		Communication or marketing		2.2 Feedback on behavior		Monitor the cost difference with the same physician who provides online and offline services and give feedback	

^a^TDF: Theoretical Domains Framework.

^b^B: barrier.

^c^EMR: electronic medical record.

^d^E: enabler.

## Discussion

### Principal Findings

This study provides novel evidence and enhances the literature on physicians’ viewpoints, particularly by applying behavioral science frameworks to understand the adoption of telemedicine. The identified factors were comprehensive, and importantly, they were modifiable and amenable to intervention—offering a solid foundation for designing targeted behavior change strategies. These frontline health care practitioners directly influence the effectiveness of telemedicine and the potential success of the digital transformation of medical institutions. To our knowledge, this was the first attempt to apply interlinked frameworks of the TDF, BCW, and BCTs to identify the influencing factors for physicians’ adoption of telemedicine and provide strategies. It enriches the understanding of individual-level factors to include wider contextual drivers, contributing to a conceptual framework for understanding multilevel influences on digital health behavior change.

There were two main findings. First, 11 of the 14 TDF domains have an impact on telemedicine adoption. The barriers comprise difficulty balancing online and offline work, technical issues, potential risks to medical quality and safety with telemedicine, and others. Enablers included that the advantages of telemedicine could guarantee the continuity of medical service. These results align with those of prior research [36,48-50]. Compared with past literature, the emotion domain and social influences domain–related factors have rarely been considered before. We added evidence of physicians’ behaviors and psychology about telemedicine adoption. Second, we presented the intervention functions, policy categories, BCTs, and potential suggestions for each barrier or enabler to ensure the proposed intervention would be feasible with high fidelity. Implementing these measures requires efforts from health administrative departments, medical institutions, departments, and health care personnel. We discuss these issues in the following sections.

### Focus on the Key Domains and Issues for Physicians’ Adoption of Telemedicine

On the one hand, the barriers to physicians’ appropriate adoption of telemedicine involved 8 TDF domains. Two domains were particularly richly described. The most evidenced domain was environmental context and resources. It comprised 9 specific barriers, among which the technique issues were the key mentioned barriers. A systematic review that researched the facilitators of and barriers to accessing telemedicine during the COVID-19 pandemic also revealed that a lack of necessary technological conditions or equipment was the main barrier to adopting telemedicine [51]. Other studies have highlighted barriers such as the lack of availability of adequate resources [37], the absence of dedicated personnel, and the paucity of professional guidelines [12]. These findings may suggest that existing mechanisms and resource conditions of telemedicine are not ready to respond to the rapid transition to a new reality of medical care delivery based on physical distancing.

Another frequently mentioned domain was memory, attention, and decision processes. The key barrier that most physicians referred to was the inefficiency caused by turnaround times in asynchronous consultations. The inability of real-time communication between physicians and patients, the inefficiency of physician-patient interactions, and poor experiences seem to be inherent shortcomings of telemedicine, which have also been mentioned several times in prior studies [52,53]. This aligns with recent research showing that approximately 70% of surveyed health care providers believe that communication difficulties between patients and health care providers result in insufficient online consultations and poor online experiences [52]. Other research has indicated that asynchronous consultations have downsides that include risks of missed urgent issues, loss of human contact and empathy, fragmentation of care, and the potential length of time one must wait for a reply. However, asynchronous consultations could be useful for some aspects of care, such as triage, monitoring the health care needs of people with long-term conditions, or reaching people who have difficulty traveling to hospitals [54]. Therefore, informed choice and flexibility are needed. It is essential to improve the design and promotion of synchronous consultations to meet the needs of different physicians and patients.

On the other hand, we also identified enablers of physicians’ adoption of telemedicine that involved 7 TDF domains. The skills and beliefs about consequences domains were particularly richly described. With respect to the skill domain, physicians believe that communication skills are the most essential for online work. Most physicians were concerned about the importance of communication, information gathering, and system operation, and they were skilled in this area of the study. They are favorable enablers of adoption. Physicians with excellent communication skills can assist with building physician-patient trust, obtaining a more accurate patient medical history and symptom information, reducing medical disputes and complaints caused by misunderstandings, and providing patient-centered health care [55,56]. However, another prior study had different findings, indicating that information access and communication challenges are particularly prominent in pediatric care, as children may struggle to express their feelings accurately [57]. Another study showed that communication skills on the screen were a challenge for some physicians in the beginning but become a natural way to communicate with patients with increasing online work experience [37].

Of the beliefs about consequences domain, the widely recognized advantages of telemedicine are enhancing accessibility and fairness. These are beneficial for the health care system, physicians, and patients [58]. Telemedicine improves physician-patient medical efficiency by reducing patient wait times in traditional hospitals, thus addressing the shortage of medical resources in China [59]. Physicians could improve their personal influence, attract more patients through the online platform, and facilitate patient management, all of which were facilitators in this study. Another advantage of telemedicine is providing patients who face geographical or time constraints with convenient access to medical services. Patients can quickly have a consultation with a physician online from the comfort of their homes, saving time and reducing costs [60], and all patients can enjoy high-quality medical services.

In addition to these benefits, compared with prior literature, we also provided further views on aspects that influence physician use, such as the emotion domain and social influences domain–related factors. These factors have rarely been considered in previous studies. Some physicians expressed negative emotions about online work. To be specific, the concerns regarding privacy breaches and the risk of disputes, anxiety stemming from fragmented communication that precludes real-time responses, and a sense of detachment fostered by an inability to see the patient dampen physicians’ enthusiasm for telemedicine, influencing their willingness to adopt it. Second, of the social influences domain, social support [61], organizational culture, and atmosphere [62] will significantly impact physicians’ thoughts, sense of responsibility, work satisfaction, and even the quality of medical service [63]. For example, several physicians mentioned that medical institutions and colleagues actively support the implementation of telemedicine services. The IT department is helpful when encountering issues with the operation of the information system. All these factors have created an atmosphere that supports and encourages the development of telemedicine, enabling physicians’ participation.

### Clarifying the Priorities of Interventions to Improve the Adoption of Telemedicine Gradually

BCTs further refine the intervention functions and policy categories of the BCW, playing a significant role in formulating specific strategies. We selected the appropriate BCTs to ensure that the proposed intervention would be feasible with high fidelity. To address these barriers and promote telemedicine participation, BCTs such as goal setting, social support, demonstration of behavior, and prompts and cues are crucial. BCTs have been widely applied in various fields of behavioral intervention, such as physical activity intervention [64] and behavioral intervention for health professionals [65]. This study demonstrated the availability and implementation of BCTs in telemedicine environments, adding to the growing body of evidence for the utility of BCTs in telemedicine health care systems.

All barriers and enablers should receive attention, and making changes requires efforts from health administrative departments, medical institutions, departments, and health care personnel. To promote active engagement in telemedicine, three key areas must be priority. First, taking fiscal measures and upgrading the technical infrastructure through hardware and software improvements are essential for a seamless telemedicine experience for both physicians and patients. Second, establishing clear guidelines and regulations that define the rights, responsibilities, and standardized operation procedures and liabilities of medical professionals in telemedicine services will instill confidence, address concerns, mitigate legal risks, and promote active involvement in telemedicine. Finally, a positive organizational atmosphere and social support play essential roles in physicians’ use, and the support of medical institutions and departments should be provided to address motivation.

### Study Limitations, Strengths, and Future Directions

There were three limitations to consider in this study. First, the participants were all from China, which may restrict the generalizability of the findings to other health care populations and telemedicine systems. For instance, an increased workload due to telemedicine was mentioned by only a few Chinese physicians, but it is quite prominently discussed in other regions [48,66,67]. Therefore, the barriers or enablers may also vary under different health care systems or work cultures. However, we used service providers in China’s health care system as our sample, expanding the literature in this area. The findings we present are quite insightful, as we recruited participants from across the different levels of medical institutions. Future research could broaden the participant scope and conduct a comparison study. Additionally, an important strength of this study is that participants were physicians with direct experience with delivering telemedicine services. This allowed us to gather context-rich insights grounded in actual clinical practice, thereby enhancing the validity and practical relevance of the results. Second, although this study provided rich qualitative insights, data were collected solely through interviews, which may be subject to recall or social desirability biases. Future studies could consider combining interviews with observations or system usage data. Third, the TDF domains face challenges regarding definition ambiguity and domain overlap, which may impact consensus, but this study achieved a high level of agreement in coding-related barriers and enabling factors and can minimize the influence of this factor on the results to the greatest extent. As thematic saturation was achieved, the results are comprehensive in terms of coverage of the issues. Even so, a notable strength of this study is the TDF framework behavior change analyses that added evidence of physicians’ behaviors and psychology about telemedicine adoption. This approach deepened our understanding of individual-level determinants to include wider contextual drivers and facilitated the development of targeted implementation strategies.

### Conclusions

As an innovative medical service model, telemedicine has excellent potential for future development. This study focuses on medical service providers’ adoption, which directly influences the efficacy of telemedicine and the potential success of the digital transformation of medical institutions. We offered vital insights into this significant topic by doing an exploration that used TDF, BCW, and BCTs. Based on these comprehensive and sequential frameworks, this study identified barriers and enablers, encompassing administrative challenges and certain clinical circumstances, including technical difficulties and suboptimal management. Notably, the barriers posed by emotional and social effects were not addressed in previous investigations. From a theoretical perspective, our study enhances the comprehension of individual-level elements and broader contextual influences. From a practical perspective, our findings are significant for promoting telemedicine implementation through focused interventions, including technological optimization, the establishment of clear guidelines, and the cultivation of a healthy organizational atmosphere.

## Appendix

Multimedia Appendix 1 Consolidated criteria for reporting qualitative studies (COREQ): 32-item checklist.

Multimedia Appendix 2 Interview questionnaire (Chinese version).

Multimedia Appendix 3 The links of TDF, BCW, and BCTs.

Multimedia Appendix 4 The BCTTv1 (English version).

Multimedia Appendix 5 Identified corresponding intervention functions, policy categories, BCTs, and implementation strategies for each barrier and enabler (complete version).

## Abbreviations

APEASE: acceptability, practicability, effectiveness, affordability, spill-over effects, and equity
BCT: behavior change technique
BCW: Behavior Change Wheel
COREQ: Consolidated Criteria for Reporting Qualitative Research
TDF: Theoretical Domains Framework

## Data Availability

The data sets used in this study are available from the corresponding author upon reasonable request.
