# Supplementary material for: Using Theory-Based Frameworks to Identify Barriers and Enablers of Physicians’ Telemedicine Adoption and Develop Intervention Strategies in China: Multicenter Qualitative Study
Source: J Med Internet Res. 2025 Sep 8;27:e73412. doi: 10.2196/73412 (PMC12455159; doi:10.2196/73412)
Supplement: Multimedia Appendix 2 [file jmir_v27i1e73412_app2.docx]

**医生访谈问卷（中文版）**

| 访谈人： | 日期： | 开始时间： | 结束时间： |
| --- | --- | --- | --- |
|  | | | |
| 医院： | 科室： | 职 称：高级 副高 中级 | |
| 年龄：(30-40]；(40-50]；(50-60]；＞60 | 性别：男 女 | 教育水平：博士 硕士 本科 | |
| 本院平台开始使用时间： 年 月 | 本院平台月工作量：＜10；[10-30]；(30-50]；(50-100]；＞100 | | |
| 本院平台工作方式：排班；报名；其他___ | 本院平台工作时间：正常上班时间内；空闲时间；其他_______ | | |
| 第三方平台开始使用时间：无/ 年 月 | 第三方平台月工作量：＜10；[10-30]；(30-50]；(50-100]；＞100 | | |
| 医生姓名： | 联系电话： | | |

xx医生您好，感谢您在百忙之中参与我们的访谈。我是来自北京大学第三医院的研究人员，我们目前正在开展一项关于医生使用互联网诊疗的认知与行为相关研究，本次访谈旨在了解您在临床实践中关于互联网诊疗的态度、看法，以及使用情况等，以便我们后期能够更好地优化互联网诊疗服务。访谈大约需要20分钟，您的回答将被严格保密，仅用于学术研究目的。访谈结束后，我们将给您发放200元劳务费。如您同意，我们将对访谈过程进行录音，方便后续分析。再次感谢您的参与和支持！

1. 关于互联网诊疗您有哪些了解？（知识）
2. 您觉得哪些情况适合/不适合使用互联网诊疗(医患双方)？（知识）
3. 您觉得互联网诊疗有哪些好处？（关于后果的信念）
4. 您觉得互联网诊疗有哪些坏处？（关于后果的信念）
5. 您觉得通过互联网诊疗能实现哪些目的？（目标）
6. 您愿意通过互联网诊疗接诊患者吗？（意图）
7. 您觉得互联网诊疗能解决患者问题吗？（意图）
8. 您知道如何开展和使用互联网诊疗吗？（技能）
9. 您觉得医生开展互联网诊疗需要具备哪些技能，您觉得您是擅长的吗？（技能）
10. 您在开展互联网诊疗时需要其他人帮您吗？是谁帮助您的，是如何帮助您的？（关于能力的信念）
11. 您觉得互联网诊疗会给患者带来不同吗？（乐观主义）
12. 您以后会加大、减小还是保持互联网诊疗在您总体医疗服务中的占比，为什么？ （乐观主义）
13. 您在开展互联网诊疗时是否存在难以记住、注意，或者是决策方面的困难吗？（记忆、注意力和决策过程）
14. 您在开展互联网诊疗时总体情绪感受是怎样的？（情绪）
15. 不同医生群体会对互联网诊疗有不同的需求吗？您观察到不同年龄、性别、学历的医生对互联网诊疗的态度有什么不同？（社会/专业角色和身份）
16. 您觉得自己具备提供互联网诊疗相关的能力吗？（关于能力的信念）
17. 您觉得哪些因素能激励您更好地投入到互联网诊疗中？ （强化）
18. 您所在的机构及同事对于互联网诊疗持什么样的看法？他们的观点会影响您使用互联网诊疗吗？（社会影响）
19. 您觉得支持的和不支持的医生群体之间有什么区别，具体是什么？（社会影响）
20. 哪些环境和资源会影响您提供互联网诊疗服务？这些对于成功开展互联网诊疗有多大影响？（环境背景和资源）
21. 您觉得成功开展互联网诊疗服务还需要作出哪些改变？ （行为调控）
22. 如何才能让互联网诊疗过程更轻松愉快？（行为调控）
