# Supplementary material for: Using Theory-Based Frameworks to Identify Barriers and Enablers of Physicians’ Telemedicine Adoption and Develop Intervention Strategies in China: Multicenter Qualitative Study
Source: J Med Internet Res. 2025 Sep 8;27:e73412. doi: 10.2196/73412 (PMC12455159; doi:10.2196/73412)
Supplement: Multimedia Appendix 3 [file jmir_v27i1e73412_app3.pdf]

Linking COM-B and TDF to BCW intervention functions

The COM-B and TDF identify what needs to shift for the desired behaviour to be achieved and therefore what to target in an intervention. The BCW identifies intervention functions and supporting policies likely to be effective in bringing about change. The links between COM-B, TDF and the intervention functions, identified by a group of experts in a consensus exercise are shown in Tables 2.2 and 2.3. For each COM-B component or TDF domain identified as relevant in bringing about the desired change in the target behaviour, Table 2.2 shows which intervention function is likely to be effective in bringing about that change.

Table 2.2 Links between COM-B, TDF and intervention functions

COM-B	TDF	Intervention functions
Physical capability	Physical skills	Training
Psychological capability	Knowledge	Education
	Cognitive and interpersonal skills	Training
	Memory, attention and decision processes	Training<br>Environmental restructuring<br>Enablement
	Behavioural regulation	Education<br>Training<br>Modelling<br>Enablement

Table continued.

Reflective motivation	Professional/social role and identity	Education<br>Persuasion<br>Modelling
	Beliefs about capabilities	Education<br>Persuasion<br>Modelling<br>Enablement
	Optimism	Education<br>Persuasion<br>Modelling<br>Enablement
	Beliefs about consequences	Education<br>Persuasion<br>Modelling
	Intentions	Education<br>Persuasion<br>Incentivisation<br>Coercion<br>Modelling
	Goals	Education<br>Persuasion<br>Incentivisation<br>Coercion<br>Modelling<br>Enablement
Automatic motivation	Reinforcement	Training<br>Incentivisation<br>Coercion<br>Environmental restructuring
	Emotion	Persuasion<br>Incentivisation<br>Coercion<br>Modelling<br>Enablement

Physical opportunity	Environmental context and resources	Training<br>Restriction<br>Environmental restructuring<br>Enablement
Social opportunity	Social influences	Restriction<br>Environmental restructuring<br>Modelling<br>Enablement

Table 2.9 Matrix of links between intervention functions and policy categories

	**Intervention functions**								
**Policy Categories**	**Education**	**Persuasion**	**Incentivisation**	**Coercion**	**Training**	**Restriction**	**Environmental restructuring**	**Modelling**	**Enablement**
Communication/ marketing	■	■	■	■				■	
Guidelines	■	■	■	■	■	■	■		■
Fiscal measures			■	■	■		■		■
Regulation	■	■	■	■	■	■	■		■
Legislation	■	■	■	■	■	■	■		■
Environ./ Social planning							■		■
Service provision	■	■	■	■	■			■	■

Linking intervention functions with BCTs

The first step is to consider all the BCTs that could be considered for any particular function. BCTs appropriate for each function, as judged by a consensus of four experts in behaviour change, are shown in Table 3.3. When considering BCTs, it is essential to be guided by the definition not by the label (see Appendix 4). The next step is to narrow the 'long list' of BCTs down to ones that are most likely to be appropriate for the situation in which you are intervening. In addition to considering the APEASE criteria (Table 1), another way of narrowing down the list is to first consider BCTs used most frequently[5] before considering less frequently used BCTs. These are also shown in bold in Table 3.3. It should be noted that the BCTs in BCTTv1 have been identified mostly from interventions directly targeting individuals and so are represented more frequently by some intervention functions (especially 'enablement') than others (most notably 'restriction' which does not feature in BCTTv1. Linking the BCW to BCTs has drawn attention to the need to develop taxonomies of BCTs across all the intervention functions. This will require detailed analyses of interventions targeting community, organisational and population levels in much the same way as has been done for interventions directly targeting individuals.

[5] BCTs were identified in a study using the BCT Taxonomy v1 to identify intervention content and defined as frequently used if they appeared in 16 or more of 40 intervention descriptions included in the study [72].

Table 3.3 Linking intervention functions to BCTs

Intervention function	Individual BCTs
Education	**Most frequently used BCTs:**<br>• **Information about social and environmental consequences**<br>• **Information about health consequences**<br>• **Feedback on behaviour**<br>• **Feedback on outcome(s) of the behaviour**<br>• **Prompts/cues**<br>• **Self-monitoring of behaviour**<br><br>Less frequently used BCTs:<br>• Biofeedback<br>• Self-monitoring of outcome(s) of behaviour<br>• Cue signalling reward<br>• Satiation<br>• Information about antecedents<br>• Re-attribution<br>• Behavioural experiments<br>• Information about emotional consequences<br>• Information about others' approval
Persuasion	**Most frequently used BCTs:**<br>• **Credible source**<br>• **Information about social and environmental consequences**<br>• **Information about health consequences**<br>• **Feedback on behaviour**<br>• **Feedback on outcome(s) of the behaviour**<br><br>Less frequently used BCTs:<br>• Biofeedback<br>• Re-attribution<br>• Focus on past success<br>• Verbal persuasion about capability<br>• Framing/reframing<br>• Identity associated with changed behaviour<br>• Identification of self as role model<br>• Information about emotional consequences<br>• Salience of consequences<br>• Information about others' approval<br>• Social comparison

Table continued.

**Incentivisation**	**Most frequently used BCTs:**<br>• **Feedback on behaviour**<br>• **Feedback on outcome(s) of behaviour**<br>• **Monitoring of behaviour by others without evidence of feedback**<br>• **Monitoring outcome of behaviour by others without evidence of feedback**<br>• **Self-monitoring of behaviour**<br><br>Less frequently used BCTs:<br>• Paradoxical instructions<br>• Biofeedback<br>• Self-monitoring of outcome(s) of behaviour<br>• Cue signalling reward<br>• Remove aversive stimulus<br>• Reward approximation<br>• Rewarding completion<br>• Situation-specify reward<br>• Reward incompatible behaviour<br>• Reduce reward frequency<br>• Reward alternate behaviour<br>• Remove punishment<br>• Social reward<br>• Material reward<br>• Material reward (outcome)<br>• Self-reward<br>• Non-specific reward<br>• Incentive<br>• Behavioural contract<br>• Commitment<br>• Discrepancy between current behaviour and goal<br>• Imaginary reward

**Coercion**	**Most frequently used BCTs:**<br>• **Feedback on behaviour**<br>• **Feedback on outcome(s) of behaviour**<br>• **Monitoring of behaviour by others without evidence of feedback**<br>• **Monitoring outcome of behaviour by others without evidence of feedback**<br>• **Self-monitoring of behaviour**<br><br>Less frequently used BCTs:<br>• Biofeedback<br>• Self-monitoring of outcome(s) of behaviour<br>• Remove access to the reward<br>• Punishment<br>• Behaviour cost<br>• Remove reward<br>• Future punishment<br>• Behavioural contract<br>• Commitment<br>• Discrepancy between current behaviour and goal<br>• Incompatible beliefs<br>• Anticipated regret<br>• Imaginary punishment
**Training**	**Most frequently used BCTs:**<br>• **Demonstration of the behaviour**<br>• **Instruction on how to perform a behaviour**<br>• **Feedback on the behaviour**<br>• **Feedback on outcome(s) of behaviour**<br>• **Self-monitoring of behaviour**<br>• **Behavioural practice/rehearsal**<br><br>Less frequently used BCTs:<br>• Biofeedback<br>• Self-monitoring of outcome(s) of behaviour<br>• Habit formation<br>• Habit reversal<br>• Graded tasks<br>• Behavioural experiments<br>• Mental rehearsal of successful performance<br>• Self-talk<br>• Self-reward

Table continued.

**Restriction**	*No BCTs in BCTTv1 are linked to this intervention function because they are focused on changing the way that people think, feel and react rather than the way the external environment limits their behaviour.*
**Environmental restructuring**	**Most frequently used BCTs:**<br>• **Adding objects to the environment**<br>• **Prompts/cues**<br>• **Restructuring the physical environment**<br><br>Less frequently used BCTs:<br>• Cue signalling reward<br>• Remove access to the reward<br>• Remove aversive stimulus<br>• Satiation<br>• Exposure<br>• Associative learning<br>• Reduce prompt/cue<br>• Restructuring the social environment
**Modelling**	**Most frequently used BCTs:**<br>• **Demonstration of the behaviour**
**Enablement**	**Most frequently used BCTs:**<br>• **Social support (unspecified)**<br>• **Social support (practical)**<br>• **Goal setting (behaviour)**<br>• **Goal setting (outcome)**<br>• **Adding objects to the environment**<br>• **Problem solving**<br>• **Action planning**<br>• **Self-monitoring of behaviour**<br>• **Restructuring the physical environment**<br>• **Review behaviour goal(s)**<br>• **Review outcome goal(s)**

	Less frequently used BCTs:<br>• Social support (emotional)<br>• Reduce negative emotions<br>• Conserve mental resources<br>• Pharmacological support<br>• Self-monitoring of outcome(s) of behaviour<br>• Behaviour substitution<br>• Overcorrection<br>• Generalisation of a target behaviour<br>• Graded tasks<br>• Avoidance/reducing exposure to cues for the behaviour<br>• Restructuring the social environment<br>• Distraction<br>• Body changes<br>• Behavioural experiments<br>• Mental rehearsal of successful performance<br>• Focus on past success<br>• Self-talk<br>• Verbal persuasion about capability<br>• Self-reward<br>• Behavioural contract<br>• Commitment<br>• Discrepancy between current behaviour and goal<br>• Pros and cons<br>• Comparative imagining of future outcomes<br>• Valued self-identity<br>• Framing/reframing<br>• Incompatible beliefs<br>• Identity associated with changed behaviour<br>• Identification of self as role model<br>• Salience of consequences<br>• Monitoring of emotional consequences<br>• Anticipated regret<br>• Imaginary punishment<br>• Imaginary reward<br>• Vicarious consequences
