# Supplementary material for: Using Theory-Based Frameworks to Identify Barriers and Enablers of Physicians’ Telemedicine Adoption and Develop Intervention Strategies in China: Multicenter Qualitative Study
Source: J Med Internet Res. 2025 Sep 8;27:e73412. doi: 10.2196/73412 (PMC12455159; doi:10.2196/73412)
Supplement: Multimedia Appendix 5 [file jmir_v27i1e73412_app5.docx]

| Identified corresponding intervention functions, policy categories, BCTs, and implementation strategies for each barrier and enabler (complete version). | | | | | |
| --- | --- | --- | --- | --- | --- |
| TDF^a^ domains | Themes of the barriers and enablers to use telemedicine  (abbreviated version) | Intervention functions | Policy categories | BCTs | Proposed implementation strategies |
| Knowledge | Limited awareness and familiarity with the app’s functionalities(B^b^) | Education | Communication or marketing | 4.1 Instruction on how to perform the behavior, 7.1 Prompts/cues | Disseminate advantages and applicable scenarios for healthcare providers and patients via official WeChat |
|  | Operational challenges of patients (B) |  |  | 4.1 Instruction on how to perform the behavior, 6.1 Demonstration of the behavior, 7.1 Prompts/cues | Provide instructions and interactive guides on how to use telemedicine for physician‒patient interactions via official WeChat |
|  | Limited applicability of telemedicine (B) |  | Regulation | 1.1 Goal setting (behavior),  1.3 Goal setting (outcome) | Understand each department and disease traits, schedule suitable for online work |
| Memory, attention, and decision processes | Inefficiencies caused by turnaround times in asynchronous consultations(B) | Environment restructuring | Guidelines | 7.1 Prompts/cues | Consider displaying physician’s availabilities based on work schedules, e.g. 12:00–13:00 and 20:00–22:00 |
|  | Limited ability to articulate conditions(B) |  |  |  | Provide a standardized medical history collection template to patients before telemedicine sessions |
|  | Others consult on behalf of patients(B) |  |  |  | Provide a standardized medical history collection template, and family members can assist in filling out |
|  | Enable real-time collection information(E^c^) | Enablement | Service Provision | 3.2 Social support (practical) | IT departments enhance the functionality by integrating AI to assist for physician decision-making |
| Skills | Clinical capabilities(E) | Training | Guidelines | 4.1 Instruction on how to perform the behavior,  6.1 Demonstration of the behavior,  8.1 Behavioral practice/rehearsal | Implement training programs for physicians on the basic operations of online consultations, including behavioral demonstrations, and practice/rehearsal sessions |
|  | Clinical experience(E) |  |  |  |  |
|  | Communication skills(E) |  |  |  |  |
|  | Information gathering skills(E) |  |  |  |  |
|  | Adaptability (E) |  |  |  |  |
|  | System operation skills (E) |  |  |  |  |
| Social influences | Lack of collaboration within professionals(B) | Enablement | Guidelines | 1.1 Goal setting (behavior), 1.4 Action planning | Develop objectives and standardized procedures for physicians’ online medical services |
|  | Insufficient customer service personnel(B) |  | Service Provision | 3.1 Social support (unspecified), 3.2 Social support (practical), 3.3 Social support (emotional) | Establish professional medical teams and nonmedical support staff for physicians |
|  | Support by medical institutions and colleagues(E) |  | Service Provision |  | Assign a team of customer service representatives to provide support to both physicians and patients |
|  | IT department is helpful when encountering issues(E) |  |  |  | Foster a positive organizational climate, and provide support to physicians |
| Environmental context  and resources | Not a well-established pricing mechanism(B) | Enablement | Fiscal measures | 1.3 Goal setting (outcome), 3.2 Social support (practical) | Set pricing standards and adjust the charging scope: link professional titles to medical service fees |
|  | Inadequate medical insurance mechanisms(B) |  |  |  | Increase financial investment to promote real-time and cross-region settlement functionalities for medical insurance |
|  | Restricted scope of telemedicine services(B) |  | Regulation | 1.1 Goal setting (behavior), 1.3 Goal setting (outcome) | Incorporate more suitable online consultation services, such as nursing consultation fees |
|  | Imperfect medical institution management mechanism(B) |  |  |  | Strategically allocate resources such as personnel, finances, and materials to coordinate telemedicine services |
|  | Imbalance workload of online and offline (B) |  |  |  | Establish reasonable online and offline work objectives for physicians to enact a balanced distribution of tasks |
|  | EMRs^d^ are not accessible across different institutions(B) | Environment restructuring | Environmentalor social planning | 12.1 Restructuring the physical environment, 12.5 Adding objects to the behavior | Boost technical or financial support to strive for cross-regional sharing of EMRs |
|  | Inconsistent system performance(B) |  |  |  | Boost technical support to enhance system functionality and environment reconstruction or optimization |
|  | Network delay(B) |  |  |  | Boost technical support to optimize network environment |
|  | Inadequate platform usability (B) |  |  |  | Boost technical support to enhance platform functionality diversity, focus on age-appropriate adaptations |
|  | Lack of telemedicine venues(B) |  |  |  | Boost financial support to rebuild the physical environment to provide specialized telemedicine facilities |
| Social/ professional role and identity | Limited acceptance among senior physicians (B) | Persuasion | Communication or marketing | 2.2 Feedback on behavior | Promote information about telemedicine for senior physicians, persuade them to conduct and feedback |
|  | Limited willingness among physicians with senior titles (B) |  |  |  | Inform senior physicians benefits of telemedicine, persuade them to conduct and feedback |
| Optimism | Convinced of telemedicine’s benefits are willing to devote more effort (E) | Education | Communication or marketing | 15.3 Focus on past,  2.2 Feedback on behavior | Advise physicians to recall successful experiences of telemedicine, monitor physicians’ behavior and feedback |
| Beliefs about  consequences | Potential risks to medical quality(B) | Education | Guidelines | 4.4 Behavioral experiments, 4.1 Instruction on how to perform the behavior | Collect online adverse events and provide recommendations on how to ensure medical safety and quality |
|  | Low efficiency in online interactions(B) |  |  | 6.1 Demonstration of the behavior | Provide customized response templates to physicians that improve the efficiency of physician‒patient interaction |
|  | Affect the physician‒patient experience(B) |  | Communication or marketing | 13.2 Framing/reframing | Propose practices that can improve the physician‒patient experience |
|  | Improve the accessibility of medical service(E) |  |  | 9.1 Credible source, 6.1 Demonstration of the behavior | Use authoritative information sources to provide references or imitations for physicians |
|  | Guarantee the continuity of medical services(E) |  |  | 6.1 Demonstration of the behavior, 8.3 Habit formation, | Demonstrate to physicians the benefits of telemedicine for continuous care and recommend using it to develop "habitual behaviors" |
|  | Improve physicians’ influence(E) |  |  | 6.2 Social comparison, 6.3 Information about others’ approval | Display to physicians the performance metrics of successful telemedicine using individuals, and compare these metrics with the physician’s personal performance |
|  | Attract more patients(E) |  |  | 9.1 Credible source, 6.1 Demonstration of the behavior | Use authoritative information sources to inform physicians that compelling patient attraction is achievable through online channels |
|  | Facilitate physicians’ management of patients(E) |  |  | 6.2 Social comparison, 6.3 Information about others' approval | Demonstrate to physicians the practices and advantages of online patient management, comparing these with the physician's owns |
|  | Reduce the patients’ burdens(E) |  |  | 2.2 Feedback on behavior | Monitor the cost difference with the same physician who provides online and offline services and give feedback |
| Intentions | Scheduled at the department of online work(B) | Persuasion | Regulation | 10.3 Non-specific reward, 10.6 Non-specific incentive | Encourage physicians engage in telemedicine, and provide material and nonmaterial incentives to physicians |
| Emotion | Concerned(B) | Persuasion | Service Provision | 3.3 Social support (emotional) | Encourage physicians to enhance their recognition and trust in online consultations, providing emotional support and assistance to foster a positive attitude toward telemedicine |
|  | Anxiety(B) |  |  |  |  |
|  | Detachment(B) |  |  |  |  |
|  | Accomplishment(E) |  |  |  |  |
|  | Relaxed(E) |  |  |  |  |
|  | Calm(E) |  |  |  |  |
|  | Enjoyable(E) |  |  |  |  |
| Reinforcement | Increase the proportion of online workload assessment (E) | Incentivization | Regulation | 2.2 Feedback on behavior, 10.3 Non-specific reward, 10.6 Non-specific incentive | Monitor physicians’ telemedicine behavior, provide material and nonmaterial incentives: boost online workload’s weight in performance evaluations |
|  |  | Coercion |  | 10.11 Future punishment | Inform physicians that if failure to meet outcomes may result in penalties or loss of future rewards |
|  | Link online work evaluation to the promotion of professional titles(E) | Incentivization |  | 2.2 Feedback on behavior, 10.3 Non-specific reward, 10.6 Non-specific incentive | Monitor physicians’ telemedicine, provide material and nonmaterial rewards: link online evaluations to promotions and titles |
|  |  | Coercion |  | 10.11 Future punishment | Inform physicians that if failure to meet outcomes may result in penalties or loss of future rewards |
|  | Links patient evaluation to work assessment(E) | Incentivization |  | 2.2 Feedback on behavior, 10.3 Non-specific reward, 10.6 Non-specific incentive | Monitor physicians’ telemedicine behavior, provide material and nonmaterial reward commitments: link patient evaluations with work evaluations |
|  |  | Coercion |  | 10.11 Future punishment | Inform physicians that failure to meet outcomes may result in penalties or loss of future rewards |

^a^TDF: Theoretical Domains Framework.

^b^B: barrier.

^c^E: enabler.

^d^EMR: electronic medical record.
